# The efficacy of immune checkpoint inhibitors in anaplastic lymphoma kinase‐positive non‐small cell lung cancer

**DOI:** 10.1111/1759-7714.13195

**Published:** 2019-09-11

**Authors:** Ja Yoon Heo, Changhee Park, Bhumsuk Keam, Chan‐Young Ock, Miso Kim, Tae Min Kim, Dong‐Wan Kim, Se Hyun Kim, Yu Jung Kim, Jong Seok Lee, Dae Seog Heo

**Affiliations:** ^1^ Department of Internal Medicine Seoul National University Hospital Seoul Korea; ^2^ National Health Insurance Service Ilsan Hospital Goyang Korea; ^3^ Cancer Research Institute Seoul National University Seoul Korea; ^4^ Department of Internal Medicine Seoul National University Bundang Hospital Seongnam Korea

**Keywords:** Anaplastic lymphoma kinase, immune checkpoint inhibitors, non‐small cell lung cancer

## Abstract

**Background:**

Despite recent advances in treating non‐small cell lung cancer (NSCLC) with immune checkpoint inhibitors (ICIs), their role in ALK‐positive NSCLC patients is unclear. We investigated the efficacy of ICIs in patients with ALK‐positive NSCLC.

**Methods:**

Between 2011 and 2018, a total of 14 ALK‐positive NSCLC patients treated with ICIs were evaluated retrospectively. Clinicopathologic features including age, PD‐L1 expression, and treatment outcomes were analyzed. RNA expression level and cytolytic activity by ALK positivity were analyzed using The Cancer Genome Atlas (TCGA) and National Cancer Center Research Institute (NCCRI) data sets.

**Results:**

A total of 13 patients (92.9%) received ALK inhibitors. Patients received a median of three (range 2–8) courses of therapy. The study included nine patients (64.3%) who were PD‐L1‐high (>50%) and four (28.6%) who were PD‐L1‐low (<50%). The objective response rate was 14.3% (2/14). The median progression‐free survival time was 2.18 months (95% confidence interval [CI] 1.13 months‐not reached [NR]). The median overall survival time was 5.67 months (95% CI 3.00 months‐NR). RNA expression levels of CD274 were similar between the ALK‐positive and negative groups in both TCGA and NCCRI datasets. RNA levels of CD8A in both TCGA and NCCRI data sets were nonsignificantly lower in the ALK‐positive group. Cytolytic activity scores including interferon‐γ‐related response were lower in the ALK‐positive group in the NCCRI but not TCGA dataset.

**Conclusions:**

Despite high PD‐L1‐positive rates, ICIs show limited efficacy in ALK‐positive NSCLC. Decreased interferon‐γ‐related response may underlie these findings.

## Key points

From retrospective review of 14 ALK‐positive NSCLC patients in South Korea treated with ICIs, the role of ICI in ALK‐positive NSCLC was navigated. Despite high PD‐L1‐positive rates, ICIs show limited efficacy (ORR 14.3%; median PFS 2.18 months; median OS 5.67 months). From TCGA and NCCRI dataset analysis, decreased interferon‐γ‐related response may underlie these findings.

## Introduction

Anaplastic lymphoma kinase (ALK) rearrangement has been observed in 3%–5% of non‐small cell lung cancers (NSCLCs).^1^, ^2^ ALK‐positive NSCLC is a distinct subgroup, and ALK tyrosine kinase inhibitors (TKIs) have been shown to prolong survival.^3^, ^4^, ^5^ Crizotinib, the first ALK inhibitor, showed superior outcomes compared to standard cytotoxic chemotherapy in advanced ALK‐positive NSCLC.^3^ Subsequently, ceritinib also showed its superiority to cytotoxic chemotherapy, and alectinib proved its efficacy compared to crizotinib.^4^, ^5^

Although ALK TKIs yield high response rates (>60%) in advanced ALK‐positive NSCLC,^3^, ^4^, ^5^ patients who initially respond to these agents eventually experience resistance and disease progression.^6^ For patients who progress after ALK TKI therapy, initial cytotoxic chemotherapy regimens, which are used for first‐line treatment of NSCLC (e.g., carboplatin/paclitaxel), are recommended.^6^

Recently, immune checkpoint inhibitors (ICIs) targeting programmed death 1 (PD‐1) and its ligand (PD‐L1) have demonstrated impressive results in NSCLC.^7^, ^8^, ^9^ NSCLC harboring an ALK rearrangement is reported to be significantly associated with PD‐L1 expression.^10^ Preclinical data suggest that ALK TKIs enhance antitumor immunity by downregulating PD‐L1, implying possibilities for anti‐PD‐1/PD‐L1 antibodies as therapeutic options for progression following ALK TKI therapy.^11^ However, data from the US Flatiron Health database and clinical trials show poor results of ICIs for ALK‐positive NSCLC.^12^ Clinical trials evaluating the combination of ICIs with ALK TKIs in advanced NSCLC are ongoing (NCT02574078, NCT01998126, NCT02393625, NCT02013219), but clinical data of patients with ALK‐positive NSCLC still remain insufficient.

Overall, the role of ICIs in treating ALK‐positive NSCLC is not yet elucidated. Therefore, in this study, we conducted a retrospective analysis to investigate the efficacy of ICIs in treating patients with ALK‐positive NSCLC.

## Methods

### Patients and data collection

We reviewed the records of patients diagnosed with ALK‐positive NSCLC and receiving treatments to block PD‐1/PD‐L1 at Seoul National University Hospital (SNUH) and Seoul National University Bundang Hospital (SNUBH) between 1 January 2011 and 31 August 2018. We identified 14 ALK‐positive NSCLC patients who received ICIs in their disease course. Clinicopathologic features including age, sex, smoking history, and treatment outcomes were reviewed from their medical history.

### Detection of ALK rearrangement

ALK positivity was defined by the ALK break‐apart fluorescence in situ hybridization (FISH) assay as evaluated by three experienced pathologists. FISH was performed on formalin‐fixed paraffin‐embedded tumor tissues using a break‐apart probe specific to the ALK locus (Vysis LSI ALK Dual Color, break‐apart rearrangement probe; Abbott Molecular, Abbott Park, IL, USA) according to the manufacturer's instructions, as described in previous reports.^13^ ALK FISH was performed from biopsy specimens prior to ALK TKI treatment.

### Immunohistochemistry of PD‐L1

Immunohistochemistry was performed using the following monoclonal antibodies against PD‐L1: 22C3 (Dako, Glostrup, Denmark), E1L3N (Cell Signaling Technology, Danvers, MA, USA), and SP263 (Ventana, Tucson, AZ, USA). Staining with clone 22C3 was performed with the corresponding Dako pharmDx assay. Staining with clone SP263 on the Ventana Benchmark Ultra platform was performed with the SP263 Ventana assay. In this study, a score of 50% was defined as “PD‐L1‐high” and a score of less than 50% or equal to 50% as “PD‐L1‐low”. Details of PD‐L1 immunohistochemistry profiles are described in Table S1.

### Open database analyses of RNA expression

Two publicly available data sets were used; one was lung adenocarcinoma of The Cancer Genome Atlas (TCGA)^14^ and the other was generously provided by the authors from an earlier publication which is referred to as the National Cancer Center Research Institute (NCCRI) dataset (accession number GSE31210).^15^ Level 3 data of TCGA lung adenocarcinoma from the UCSC Cancer Browser (https://genome-cancer.ucsc.edu) was downloaded on 3 June 2015. Both datasets included clinical information of *ALK* translocation and mRNA expression data obtained by RNAseq (TCGA using the Illumina HiSeq V2 platform) and microarray (NCCRI using the Affymetrix Human Genome U133 Plus 2.0 Array). Array data from the NCCRI dataset were normalized using the limma R package and were log2 transformed.

In these datasets, we analyzed differences in the mRNA expression of *CD8A* and *CD274* (PD‐L1) between samples with and without *ALK* translocation.^16^ We also analyzed differences in the cytolytic activity score, defined as the mean value of mRNA expression of *GZMA* (granzyme A) and *PRF1* (perforin 1).^17^ Finally, to assess interferon‐γ‐responsive gene expression, we analyzed the data sets using the “Module3_IFN_Score” gene set obtained from earlier publications, and the gene set enrichment analysis method through the GenePattern website (https://genepattern.broadinstitute.org).^18^

### Statistical analysis

Progression‐free survival (PFS) was calculated from the start date of ICI treatment to the date of disease progression by RECISTv1.1 criteria,^19^ as confirmed by imaging, death, or the last follow‐up date, if censored. Overall survival (OS) was measured from the initiation of ICI treatment until death or the last follow‐up date, if censored. Survival analyses were carried out according to the Kaplan‐Meier method with the log‐rank test. All tests were two‐sided and *P*‐values <0.05 were considered statistically significant. For survival analysis, STATA Statistical Software version 15.0 (StataCorp, College Station, TX, USA) was used for computation. Statistical analyses and image creations for RNA expression were performed using R version 3.4.3 software (R Development Core Team, https://www.r-project.org/). The Wilcoxon rank sum test was applied to analyze gene expression differences.

## Results

### Patient characteristics

We identified 14 patients with ALK‐positive NSCLC who were treated with ICIs between 2011 and 2018 at SNUH (*n* = 9) and SNUBH (*n* = 5). Baseline clinical and pathological features of these patients are summarized in Table 1.

**Table 1:** Patient characteristics

*N* = 14		N (%)
Age (years)	Median (range)	49 (25–64)
Sex	Male	10 (71.4)
	Female	4 (28.6)
Ethnicity	Asian	14 (100.0)
Smoking	Never smoker	5 (35.7)
	Current or ex‐smoker	9 (64.3)
Pathology	Adenocarcinoma	13 (92.9)
	Poorly differentiated	1 (7.1)
Prior ALK TKIs	None	1 (7.1)
	1	1 (7.1)
	2	8 (57.2)
	3	4 (28.6)
Prior lines of therapy	2	2 (14.3)
	3	7 (50.0)
	≥ 4	5 (35.7)
PD‐1 inhibitor	Nivolumab	8 (57.2)
	Pembrolizumab	5 (35.7)
PD‐L1 inhibitor	Atezolizumab	1 (7.1)
ALK rearrangement	FISH positive	14 (100.0)
PD‐L1 expression	High (>50%)	9 (64.3)
	Low (≤50%)	4 (28.6)
	Not checked	1 (7.1)

ALK, Anaplastic lymphoma kinase; ICI, immune checkpoint inhibitor; PD‐1, programmed death 1; PD‐L1, programmed death ligand; TKI, tyrosine kinase inhibitor.

A majority of patients (*n* = 13; 92.9%) had previously received and progressed on ALK TKI treatment. Patients received a median of three (range 2–8) courses of therapy. All patients received single‐agent PD‐1 or PD‐L1 inhibitors. Thirteen patients (92.9%) received the PD‐1 inhibitor nivolumab (*n* = 8; 57.2%) or pembrolizumab (*n* = 5, 35.7%). The study also included one patient (*n* = 1; 7.1%) who received atezolizumab, a PD‐L1 inhibitor. PD‐L1 expression over 50% was found in nine patients (64.3%). Four patients (28.6%) did not show PD‐L1 expression (≤50%) by immunohistochemistry.

### ICI responses

**Figure 1:**
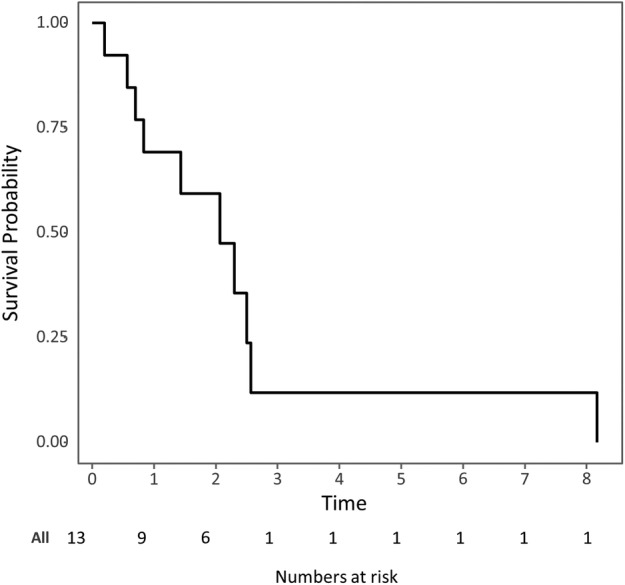
Kaplan‐Meier survival curves showing the progression‐free survival of ALK‐positive NSCLC patients treated with immune checkpoint inhibitors. ALK, anaplastic lymphoma kinase; NSCLC, non‐small‐cell lung cancer; PD‐1, programmed death 1; PD‐L1, programmed death ligand 1.

**Figure 2:**
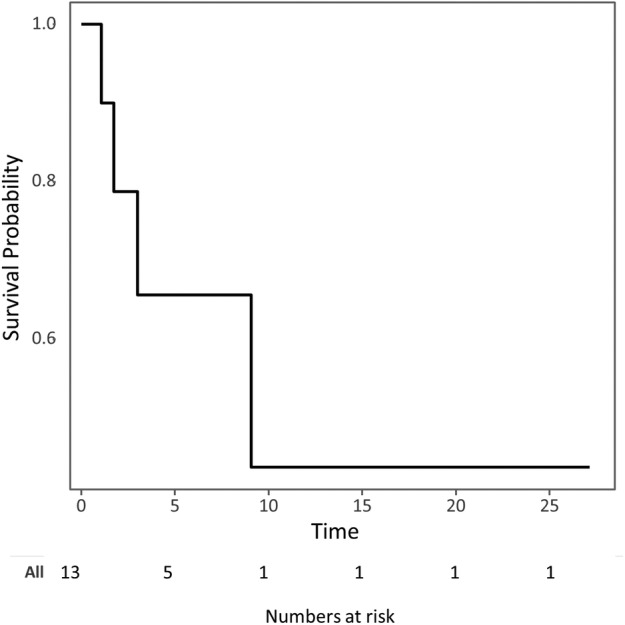
Kaplan‐Meier survival curves showing the overall survival of ALK‐positive NSCLC patients treated with immune checkpoint inhibitors. ALK, anaplastic lymphoma kinase; NSCLC, non‐small‐cell lung cancer; PD‐1, programmed death 1; PD‐L1, programmed death ligand 1.

Among patients with ALK‐positive NSCLC, the objective response rate to ICIs was 14.3% (2/14). Details are presented in Table 2. Two patients treated with pembrolizumab showed responses (duration: 8.2 and 4.1+ months). The median PFS among ALK‐positive NSCLC patients treated with ICIs was 2.18 months (95% confidence interval [CI] 1.13‐not reached [NR] months) (Fig 1). The median OS among ALK‐positive NSCLC patients treated with ICIs was 5.67 months (95% CI 3.00‐NR months) (Fig 2). The patients were followed for a median of 4.2 months (range 0.8–30.5 months).

**Table 2:** Response rates of immune checkpoint inhibitors

*N* = 14		N (%)
ICI response	CR	0 (0.0)
	PR	2 (14.3)
	SD	2 (14.3)
	PD	9 (64.3)
	Not evaluable	1 (7.1)
	ORR	2 (14.3)
PFS a (months)	Median (95% CI)	2.2 (1.1–NR)
OS b (months)	Median (95% CI)	5.7 (3.0–NR)
Follow‐up (months)	Median (range)	4.2 (0.9–30.5)

a Progression free survival from the initiation of ICI to the date of disease progression by RECISTv1.1 criteria by imaging, death, or the last follow‐up.

b Overall survival from the initiation of ICI to death or last follow‐up.

ICI, immune checkpoint inhibitor; CR, complete response; PR, partial response; SD, stable disease; PD, progressive disease; ORR, objective response rate; PFS, progression‐free survival; OS, overall survival; NR, not reached; CI, confidence interval.

### RNA expression levels and cytolytic activity score

To investigate a possible biologic explanation of our clinical observations, we used lung adenocarcinoma of TCGA and NCCRI datasets.^15^ Three out of 513 cases (0.58%) had *ALK* translocation in TCGA dataset. In the NCCRI dataset, *ALK* translocation was present in 11 out of 246 cases (4.47%).

**Figure 3:**
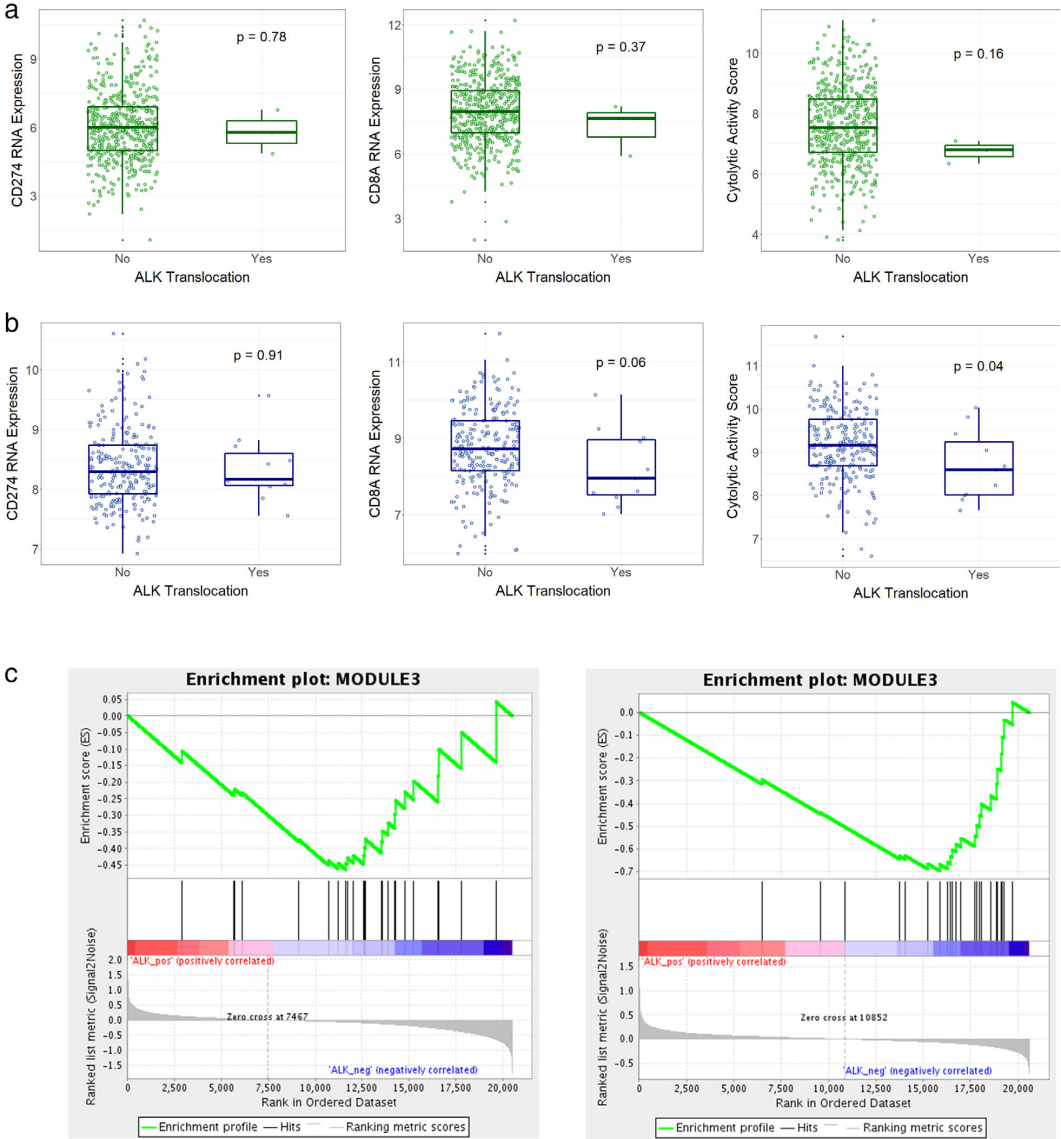
Open database analyses of RNA expression according to anaplastic lymphoma kinase translocation. Boxplots of (**a**) were created with lung adenocarcinoma of The Cancer Genome Atlas (TCGA) dataset. Boxplots of (**b**) were created with the National Cancer Center Research Institute (NCCRI) dataset. The left boxplots show RNA expression levels of CD274 (PD‐L1), and the middle boxplots show RNA expression levels of CD8A. The right boxplots show cytolytic activity scores. Statistical significance is shown with *P*‐values. (**c**) Enrichment plots were created using gene set enrichment analysis through the GenePattern website (left, TCGA dataset; right, NCCRI dataset) (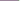) Enrichment profile, (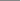) Hits, and (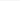) Ranking metric scores.

RNA levels of *CD274* (PD‐L1) were similar between the *ALK* translocation positive and negative groups in both TCGA and NCCRI datasets (*P*‐values = 0.780 and 0.913, respectively, Fig 3a,b, left). RNA levels of *CD8A* in both TCGA and NCCRI datasets tended to be lower in the *ALK* translocation positive group, although the tendency was not statistically significant (*P*‐values = 0.37 and 0.062, respectively) (Fig 3a,b, middle).

Next, we determined the cytolytic activity scores, which are related to local immunogenicity of the tumor microenvironment.^17^ Cytolytic activity scores were significantly lower in the *ALK* translocation positive group in the NCCRI (*P*‐value = 0.040) dataset, but not TCGA dataset (*P*‐value = 0.160) (Fig 3a,b, right). Enrichment scores of *ALK*‐positive sample gene sets of interferon‐γ,^18^ by the gene set enrichment analysis, were −0.46 and −0.70 in TCGA and NCCRI datasets (FDR = 0.67 and 0.16, respectively, Fig 3c). Collectively, these results suggest that ALK translocation tended to be associated with decreased interferon‐γ‐related responses, underlying lower response rates.

## Discussion

In this study, ALK‐positive NSCLC patients showed a low response rate of 14.3% to ICIs, and short PFS of 2.1 months and OS of 9.1 months. Although most patients exhibited high PD‐L1 expression, it did not result in higher response rates.

As a predictive biomarker for ICI treatment response, PD‐L1 expression has been widely studied in multiple clinical trials since the early time of ICI development. Both preclinical and clinical data demonstrate that ALK‐positive NSCLC is significantly associated with high expression of PD‐L1.^10^ Patients with high PD‐L1 expression, based on varying assays with different cutoffs, tend to have better responses to ICIs.^20^, ^21^ However, a positive correlation between PD‐L1 expression and the response to ICIs was not observed in ALK‐positive NSCLC patients. In this study, even though the majority of study patients had high expression levels of PD‐L1, it did not lead to a good response to ICIs.

A retrospective study including six ALK‐positive NSCLC patients found ALK rearrangement to be associated with low overall response rates to PD‐1/PD‐L1 blockade.^22^ Most recently, in the Supplementary data of a large scale phase 2 study (ATLANTIC) that included 15 ALK‐positive NSCLC patients treated with durvalumab as third‐line or later therapy, ALK‐positive NSCLC patients had a tendency of poor OS and PFS compared to EGFR‐positive patients.^23^ These findings are consistent with our study.

Preclinical data from Koh *et al*.^10^ showed that EML4‐ALK enhanced PD‐L1 expression in pulmonary adenocarcinoma via hypoxia‐inducible factor‐1α and STAT3. Similarly, induction of PD‐L1 expression by the EML4‐ALK oncoprotein and downstream signaling pathways has been reported in ALK‐positive NSCLC patients.^24^ This implies that NSCLCs harboring an ALK rearrangement are significantly associated with PD‐L1 expression.^10^, ^11^, ^24^

The effect of ALK‐TKIs on PD‐LI expression is controversial. Ota *et al*.^24^ showed that endogenous PD‐L1 expression in ALK‐positive NSCLC cells was attenuated by treatment with the specific ALK inhibitor, alectinib, or by ALK siRNA. Hong *et al*.^11^ reported that an ALK‐TKI downregulated PD‐L1 in tumor cells. Most recently, Kim *et al*.^25^ reported that PD‐L1 was expressed at higher levels in ALK inhibitor‐resistant cell lines than in the ALK inhibitor‐naïve parental cell line at the total protein, surface protein, and mRNA levels. Because of these diverse results, the clinical impact of PD‐L1 expression on ALK‐positive NSCLC tumor cells remains unclear.

Recently, the tumor mutational burden (TMB) has emerged as a predictive biomarker of ICI effectiveness.^26^ While a large‐scale clinical trial has shown that the TMB has a role in NSCLC,^27^ ALK‐positive patients have a relatively low TMB.^28^ In addition, Gainor *et al*.^22^ reported low rates of concurrent PD‐L1 expression and CD8+ tumor infiltrating lymphocytes within the tumor microenvironment of ALK‐positive NSCLC. Thus, ALK‐positive NSCLC patients may express fewer neoantigens, possibly leading to an ineffective tumor environment for ICIs. Furthermore, a decreased interferon‐γ‐related response is suggested from cytolytic activity score analysis, even though the difference in *CD274* (PD‐L1) and *CD8A* RNA expression levels was not significant.

There are several limitations to this study. Firstly, this was a retrospective analysis. Because ALK‐positive NSCLC is rare, only a small number of patients were included in the study, resulting in limited statistical power. In addition, the small study population did not allow us to perform comparisons between different groups. Secondly, we performed immunohistochemistry of PD‐L1 utilizing three different anti‐PD‐L1 antibodies (22C3, E1L3N, and SP263). Although different antibody clones can have varying cutoff levels for tumor PD‐L1 expression, our study used a unified cutoff for all antibodies. Because of a lack of tissue, we did not repeat PD‐L1 immunohistochemistry with single PD‐L1 antibodies. However, recently, the Blueprint PD‐L1 IHC Assay Comparison Project revealed that these PD‐L1 antibodies were closely aligned on tumor cell staining,^29^ minimizing this concern. Thirdly, our study was not free from tumor heterogeneity and sampling variability. Even in the same patient, PD‐L1 expression can be discordant both within and between tumor specimens.^30^ Due to invasiveness of the procedure, we could only perform a limited number of biopsies during treatment. Moreover, the timing of assessing PD‐L1 expression varied according to individual patients. As there are preclinical data that ALK‐TKIs may affect the expression level of PD‐L1,^11^, ^24^ the diversity of examination points probably influenced the results.

Despite these limitations, to our knowledge, this retrospective study is the first report in ALK‐positive NSCLC and includes the largest number of ALK‐positive NSCLC patients treated with ICIs. We believe that this report may provide useful information to clinicians regarding ALK‐positive NSCLC patients.

In conclusion, ICIs show limited effectiveness in treating ALK‐positive NSCLC even though this cancer exhibits high PD‐L1‐positive rates. Further large‐scale prospective studies are required to confirm our findings.

## Disclosure

The authors declare that there are no conflicts of interest.

## Supporting information


**Table S1.** Survival and immunohistochemistry profile of PD‐L1.
