# Supplementary material for: The efficacy of immune checkpoint inhibitors in anaplastic lymphoma kinase‐positive non‐small cell lung cancer
Source: Thorac Cancer. 2019 Sep 11;10(11):2117–23. doi: 10.1111/1759-7714.13195 (PMC6825904; doi:10.1111/1759-7714.13195)
Supplement: Supplementary file 1 — Table S1. Survival and immunohistochemistry profile of PD‐L1. [file TCA-10-2117-s001.docx]

**Supplementary table 1. Survival and Immunohistochemistry profile of PD-L1**

| Patient | Response to ICI | PFS* (months) | OS** (months) | PD-L1 antibody | Expression level | ALK TKI treated*** | duration of ALK TKI treatment | Best response to ALK TKI |
| --- | --- | --- | --- | --- | --- | --- | --- | --- |
| 01 | PD | 2.3 | 3.0 | E1L3N | 0% | Crizotinib | 0.7 | PD |
| 02 | PD | 0.9 | 0.9 | E1L3N | 50% | Crizotinib | 2.2 | PD |
|  |  |  |  |  |  | Brigatinib | 1.1 | PD |
| 03 | NE | 0.6 | 5.6 | E1L3N | 95% | Crizotinib | 2.4 | PR |
|  |  |  |  |  |  | Ceritinib | 1.2 | PD |
| 04 | PD | 0.2 | 2.1 | SP263 | 90% | Ceritinib | 41.6 | PR |
|  |  |  |  |  |  | Crizotinib | 3.1 | SD |
| 05 | PD | 0.8 | 5.7 | E1L3N | 70% | Alectinib | 14.8 | PR |
| 06 | PD | 1.4 | 1.7 | SP263 | 100% | Crizotinib | 13.3 | PR |
|  |  |  |  |  |  | Lorlatinib | 8.6 | PR |
| 07 | PD | 2.1 | 30.5 | Not checked |  | Crizotinib | 34.1 | PR |
| 08 | PD | 3.2 | 3.7 | SP263 | 5% | Crizotinib | 14.6 | SD |
|  |  |  |  |  |  | Alectinib | 1.4 | PD |
| 09 | PR | 4.1 | 4.1 | SP263 | 90% | Crizotinib | 17.3 | PR |
|  |  |  |  |  |  | Brigatinib | 14.3 | PR |
|  |  |  |  |  |  | Lorlatinib | 2.8 | SD |
| 10 | PR | 8.2 | 14.3 | 22C3 | 100% | Crizotinib | 8.4 | PR |
|  |  |  |  |  |  | Ceritinib | 5.4 | PR |
|  |  |  |  |  |  | Brigatinib | 1.4 | PD |
| 11 | SD | 2.5 | 9.1 | SP263 | 90% | Crizotinib | 17.6 | SD |
|  |  |  |  |  |  | Ceritinib | 10.0 | PR |
|  |  |  |  |  |  | Alectinib | 1.6 | PD |
| 12 | PD | 1.1 | 1.1 | 22C3 | 70% | Crizotinib | 7.8 | PR |
|  |  |  |  |  |  | Alectinib | 1.5 | PD |
| 13 | SD | 2.6 | 10.3 | SP263 | 40% | Crizotinib | 16.6 | PR |
|  |  |  |  |  |  | Ceritinib | 16.0 | SD |
|  |  |  |  |  |  | Brigatinib | 7.6 (ongoing) | PR |
| 14 | PD | 3.0 | 4.3 | SP263 | 70% | Crizotinib | 11.6 | PR |
|  |  |  |  |  |  | Alectinib | 5.3 | PR |
|  |  |  |  |  |  | Brigatinib | 0.5 | PD |

* Progression free survival from the initiation of ICI to the date of disease progression by RECISTv1.1 criteria by imaging, death, or the last follow-up

** Overall survival from the initiation of ICI to death or last follow-up

Abbreviation: ALK, anaplastic lymphoma kinase; CR, complete response; PR, partial response; SD, stable disease; PD, progressive disease; ORR, objective response rate; PFS, progression-free survival; OS, overall survival; PD-L1, programmed death ligand 1; TKI, tyrosine kinase inhibitor.

*** Treated ALK TKI is listed in order from the top.
